# Intergenerational transmission of war-related trauma assessed 40 years after exposure

**DOI:** 10.1186/s12991-019-0238-2

**Published:** 2019-08-09

**Authors:** Ivone Castro-Vale, Milton Severo, Davide Carvalho, Rui Mota-Cardoso

**Affiliations:** 1 Medical Psychology Unit, Department of Clinical Neurosciences and Mental Health, Faculty of Medicine, Universidade do Porto, Al. Prof. Hernâni Monteiro, 4200-319 Porto, Portugal; 2 Instituto de Investigação e Inovação em Saúde, Universidade do Porto, Rua Alfredo Allen 208, 4200-135 Porto, Portugal; 3 Department of Clinical Epidemiology, Predictive Medicine and Public Health, Faculty of Medicine, Universidade do Porto, Al. Prof. Hernâni Monteiro, 4200-319 Porto, Portugal; 4 Department of Medical Education and Simulation, Faculty of Medicine, Universidade do Porto, Al. Prof. Hernâni Monteiro, 4200-319 Porto, Portugal; 5 Department of Endocrinology, Diabetes and Metabolism, Centro Hospitalar Universitário Sāo Joāo, Faculty of Medicine, Universidade do Porto, Al. Prof. Hernâni Monteiro, 4200-319 Porto, Portugal

**Keywords:** Traumatic event, Parental PTSD, Intergenerational transmission, Offspring, War

## Abstract

**Background:**

The intergenerational transmission of posttraumatic stress disorder (PTSD) from parent to offspring has been suggested in the literature, but this is highly controversial. We aimed to study the association between veterans’ war exposure and lifetime PTSD and the psychological characteristics of their respective offspring, 40 years after war-related trauma.

**Methods:**

Forty-four adult offspring of veterans with PTSD and 29 offspring of veterans without PTSD were included in the study, from a total of 46 veterans. War exposure intensity, lifetime PTSD, and the general psychopathology (with Brief Symptom Inventory—BSI) of the veterans were studied, as were childhood trauma, attachment, and the general psychopathology (with BSI) of their offspring.

**Results:**

Veterans’ war exposure was associated with BSI in the offspring with regard to somatisation (*β *= 0.025; CI 0.001, 0.050), phobic anxiety (*β *= 0.014; CI: 0.000, 0.027), Global Severity Index (GSI) (*β *= 0.022; CI 0.005, 0.038), and Positive Symptom Distress Index (*β *= 0.020; CI 0.006, 0.033). The fathers’ GSI mediated only 18% of the effect of the veterans’ total war exposure on offspring’s GSI. Fathers’ war exposure was associated with offspring’s physical neglect as a childhood adversity, although non-significantly (*p *= 0.063). None of the other variables was associated with veterans’ war exposure, and veterans’ lifetime PTSD was not associated with any of the variables studied.

**Conclusions:**

The offspring of war veterans showed increased psychological suffering as a function of their fathers’ war exposure intensity, but not of their fathers’ lifetime PTSD. These results could be used to suggest that mental health support for veterans’ offspring should consider the war exposure intensity of their fathers, and not just psychopathology. This could spare offspring’s suffering if this mental health support could be delivered early on, after veterans return from war.

## Background

The experience of any traumatic event results in posttraumatic stress disorder (PTSD) development in 9.2% of individuals [1]. PTSD is a common and often chronic and debilitating condition [2]. Affected subjects have significant intrapersonal and interpersonal suffering, including within their families. Several studies have documented unhealthy family functioning in areas such as affective responsiveness, problem solving, conflict, family cohesion, marital adjustment, and offspring maltreatment [3–9].

In the case of war-related trauma, PTSD occurs in 18% of veterans [10]. War-related traumatic events (TEs) have the highest conditional risk for PTSD development [11]. PTSD is the most prevalent disorder after a TE, and the effect of combat exposure has been found to be specific to PTSD, but other consequences for health have been reported, such as depression [12]. Those deployed to war usually do so during their youngest adult years and during periods when they become fathers. Knowing that PTSD has consequences for the family (e.g. [6, 13]), war-related PTSD is to be expected to have a large impact on offspring, as they could be exposed to the consequences of their fathers’ PTSD during their development. Furthermore, the traumatic experience itself could predict interpersonal violence, and as such, have consequences for the offspring, which consequently affects parenting [14]. Several mechanisms have been described to explain the intergenerational transmission of parental traumatic experiences to offspring [15–17]. One possible mechanism could be the consequences on attachment patterns [15], as some studies indicate that attachment patterns seem to be a primary mode of trauma transmission [18, 19].

The intergenerational transmission of war-related PTSD has been suggested in the literature [20, 21], although the degree of controversy is high. PTSD is a heterogeneous disorder, and it is to be expected that the following generation is influenced by such heterogeneity at a greater level—as the traumatised parent is only one influence, among others, such as the mother’s role [4, 22]. Offspring of war veterans with PTSD have shown higher depression scores than those of war veterans without PTSD [7], and also a higher rate of aggression and anxiety than those of non-veterans [3]. However, no differences have been found regarding psychological measures between the offspring of war veterans with PTSD and a non-veteran control group [9], neither between the offspring of veterans with PTSD, veterans without PTSD, and non-veterans, including PTSD symptomatology among offspring [4]. Studies of the offspring of non-war-related TEs populations’ have also yielded contrasting results, for instance, in the case of refugees’ offspring [23, 24], of the offspring of Holocaust survivors [25–27], and of the offspring of ex-prisoners of war [22, 28]. The methodology used across studies is diverse, which naturally produces distinct results. Some studies focus on young children (e.g. [29]), others on adults (e.g. [27]), whilst some study fathers’ traumatic exposure as children (e.g. [23]), and others do not directly studied offspring, but only through their parents (e.g. [13]). In addition, others have studied fathers’ PTSD status through their offspring (e.g. [30]).

It is known that PTSD has a genetic contribution [31], and as such, the offspring of index cases may well have an enhanced risk of developing the condition, as well as other psychopathology. This has been shown in studies on parent/child simultaneous exposure [20] and also in top-down studies [26, 32, 33]. If the parents themselves are vulnerable to developing PTSD, this could then pass to their offspring, who would run the risk of developing psychopathology, which could be summed up by the direct effects of their parents’ PTSD symptoms.

Another important aspect of the intergenerational transmission of PTSD is the contribution of the father’s TE itself on the mental health of their offspring. This requires the TEs be well measured, in order to enable the study of the influence of veterans’ war traumatic experience burden on the outcome of their offspring. Rosenheck and Fontana [34] addressed this issue, but only for offspring up to the age of 16. Another study used a small sample of offspring of veterans with PTSD, with a mean age of 23 [8]. Studies on the long-term influence of veterans’ war exposure on adult offspring are lacking in the literature.

Portugal fought a war with its former colonies of Angola, Mozambique, and Guinea from 1961 to 1974. One million men participated in what has been considered to have been a guerrilla war. After more than 40 years, it is imperative to understand the consequences of the war on their offspring—even though many health issues of the veterans remain to be studied. As such, our aim was to address the following question: Are both veterans’ intensity of war exposure and lifetime PTSD status associated with their offspring’s mental health 40 years after the end of war?

Accordingly, the objective of this cross-sectional study was to study the association of war veterans’ PTSD lifetime diagnosis and war exposure intensity with the self-reported psychopathology of their adult offspring, assessed 40 years after the end of war. Offspring’s self-reported childhood adversities and attachment patterns were also studied, as these have been implicated as being possible mechanisms of intergenerational transmission of trauma and PTSD. We hypothesised that veterans’ war exposure intensity and lifetime PTSD are associated with increased childhood adversity, attachment disorganised patterns, and psychopathological symptoms in their offspring, when assessed 40 years after their fathers’ exposure to war-related trauma.

## Methods

### General procedure

This cross-sectional research is part of a larger study on the neurobiological inheritance of PTSD. This study was approved by the Ethics Committee of our University (Comissão de Ética para a Saúde do Centro Hospitalar São João/Faculdade de Medicina da Universidade do Porto, resolution number: CES-138/08), and complies with the Declaration of Helsinki and its later amendments. Having received a complete written and verbal description of the study, all those subjects who agreed to participate in the research gave their written, informed consent. In a university setting, interaction with the participants was performed solely by the same researcher and was carried out individually during just one appointment. Appointments to carry out the study were scheduled depending on the participant’s availability. No financial compensation was paid for participating in this study, although payment for transport to the university was provided. This research was carried out between December 2012 and April 2014.

### Participant selection

**Fig. 1:**
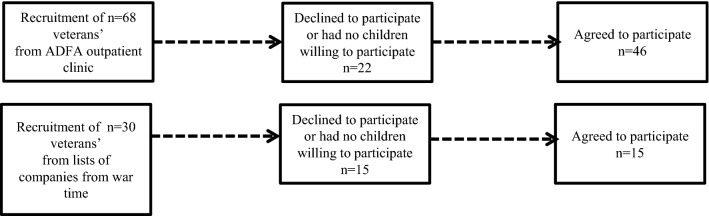
Flow chart of enrolment of the veterans’ study participants. *ADFA* Portuguese Disabled Veterans Association

The sampling procedure used two ways of selecting war veterans: (1) from an outpatient clinic of the Portuguese Disabled Veterans Association (ADFA), and (2) from three lists of veterans’ companies from wartime. ADFA veterans were first approached by their attending physician, after consultation of the clinical file for verification of eligibility to participate, based on the pre-established criteria. Others were first contacted by telephone to verify their eligibility to participate, based on the same pre-established criteria. Participants were included if they fulfilled the war-related criterion A for PTSD of the Fourth Edition of the Diagnostic and Statistical Manual of Mental Disorders (DSM-IV; [35]), and if they had children. DSM-IV war-related criterion A for PTSD was considered if the veterans had experienced, witnessed, or had been confronted with an event during the period of deployment to war which involved actual or threatened death or serious injury, or a threat to the physical integrity of themselves or others, resulting in a response of intense fear, horror, or helplessness. The general exclusion criteria for participants were the following: the presence of neurologic, infectious, or any active medical illness and also any DSM-IV psychotic, bipolar, or cognitive disorders. Participants with PTSD were also excluded if they currently had substance-related disorders. The specific control group exclusion criteria were: if the participants had ever had PTSD and any current psychiatric disorder. Figure 1 shows the flow chart of the enrolment of veterans for the study. Sixty-one male (75.4% from ADFA), Caucasian veterans from the Portuguese colonial wars agreed to participate, as did their children, with a mean age of 65.3 years (SD = 3.4, range 60–74). Women did not participate in the Portuguese colonial wars as combatants. Of the 61 veterans studied, only 46 arranged for at least one of their offspring to also participate and were included for this research. Seventy-three offspring participants accepted to participate, with a mean age of 35.7 years (SD = 4.8, range: 21–44), 60.3% of whom were females.

### Measures

Both the war veterans and their offspring were assessed using the measures described below, with the exception of the War Experiences Questionnaire, which was only answered by the veterans. The average time required to complete the interviews and questionnaires was 120 min for the veterans and 90 min for the offspring.

#### Sociodemographic data and clinical history

Socioeconomic status (SES) was measured using the Graffar Index [36], adapted for the Portuguese population [37]. This index classifies subjects into five classes, with 1 being the highest and 5 the lowest. Other sociodemographic data and the clinical history were also collected.

#### Structured Clinical Interview for DSM-IV axis I (SCID-I)

In order to determine veterans’ eligibility for the study, current and past psychiatric disorders were investigated, using SCID-I [38].

#### Clinician-Administered PTSD Scale (CAPS)

The Portuguese version of the CAPS [39, 40] was used to characterise the veterans in relation to PTSD diagnosis. Lifetime PTSD was considered if participants had DSM-IV criteria according to Blake et al.’s [41] rule (frequency ≥ 1 and intensity ≥ 2), and a total CAPS score of 50 or more. CAPS has shown high internal consistency [39] and good correlations with other measures [42, 43]. War-related TEs were assessed with CAPS Life Events Checklist (LEC) and were subsequently checked for DSM-IV A2 criterion following CAPS procedure. In our sample, the reliability (Cronbach’s alpha) of the CAPS was superior to 0.90.

#### Childhood Trauma Questionnaire-Short Form (CTQ-SF)

The CTQ-SF [44] measures five different types of maltreatment during childhood and adolescence, namely emotional abuse; physical abuse; sexual abuse; emotional neglect; and physical neglect. CTQ-SF is a self-report measure, which has been validated in a Portuguese non-clinical sample [45]. The total CTQ-SF score provides a general childhood adversity score—and not just for traumatic events. The questionnaire contains 28 items, which enquire about specific maltreatment experiences. Items are classified into a five-point Likert scale, according to the frequency of exposure to that specific experience. This questionnaire has shown good results with regard to time stability, as well as divergent and convergent validity [44, 46]. In this study, we used each subscale and the total CTQ-SF score to study the offspring.

#### Revised Adult Attachment Scale (RAAS)

Offspring’s attachment dimensions were studied using the RAAS [47, 48]. RAAS consists of 18 items relating to the subjects’ general orientation towards close relationships, which are rated on a 5-point Likert scale. This scale contains three dimensions: anxiety, close, and depend. Close and depend dimensions are positively correlated and can be grouped as close–depend. In our study, the Portuguese version was used, which has been validated for the Portuguese population [49].

#### War Exposure Questionnaire (WEQ)

In order to characterise and quantify the different war-related experiences of the fathers, we designed the WEQ (Castro-Vale and Maia 2012, unpublished), which was adapted to the specificities of the guerrilla war in which the participants were involved in. This questionnaire was adapted from the “Severity of Exposure Index”, which is used for the same purpose, and has good internal consistency (*α* = 0.79) [50]. The WEQ comprises 38 items, including: Have you been tortured? Have you participated in combat situations in which you could have lost your life? and Have you been sleepless for several days? The total sum of positively answered questions provided a total WEQ score (ranging from 0 to 38)—which represents war exposure and was used as a surrogate for war severity. In our sample, the reliability (Cronbach’s alpha) of the total WEQ score was 0.81.

#### Brief Symptom Inventory (BSI)

Participants’ current general psychopathology was evaluated using the BSI [51], which is a self-rating scale and details nine symptom dimensions: somatisation; obsessive–compulsive; interpersonal sensitivity; depression; anxiety; hostility; phobic anxiety; paranoid ideation; and psychoticism. This scale includes three global indices of distress: The Global Severity Index (GSI), which refers to the level of recent self-reported psychological distress and is calculated as the mean value of all item responses; the Positive Symptom Total (PST), which is the number of items scored above zero, and the Positive Symptom Distress Index (PSDI), which corresponds to the overall intensity of symptoms and is calculated as the average score of all items scored above zero. BSI was validated for the Portuguese population showing good construct validity and a Cronbach’s alpha between 0.62 and 0.80 [52]. In our study, the Cronbach’s alpha was 0.98 for GSI, and it varied between 0.81 for paranoid ideation and 0.90 for somatisation and depression for the symptoms dimensions. The following dimensions of BSI showed a right-skewed distribution: somatisation; depression; hostility; and paranoid ideation. For these dimensions, we applied a natural log transformation to obtain a simetric distribution. All the other variables were normally distributed.

### Data analysis

**Fig. 2:**
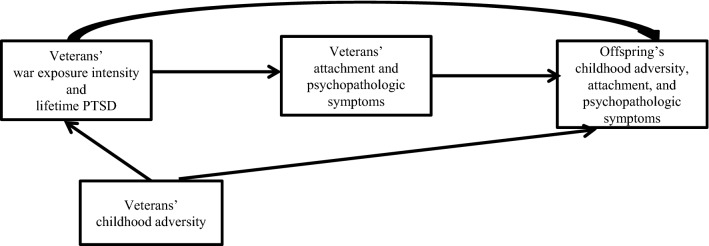
The relationships studied between the exposure variables (veterans’ war exposure intensity and lifetime PTSD) and the outcome variables (offspring’s childhood adversity, attachment, and psychopathologic symptoms)

Sociodemographic characteristics differences between the groups were analysed with *t* tests for continuous variables, and Chi-square or Fisher’s exact tests for categorical variables. The Shapiro test was used to check whether the data were normally distributed. In the scores of the CTQ-SF and BSI, some participants did not answer all the items, and thus, we used simple mean imputation. CTQ-SF had 78.6% of the items with no missing answers, and 21.4% of the items had only one missing answer. BSI had 90.6% of the items with no missing answers, and 9.4% of the items had one to three missing answers. Considering that 52% of the veterans in our sample had more than one child, the model used was a mixed effects model, with a random effect by family, in order to study the association between offspring’s psychological variables and the veterans’ variables. The outcome variables of the model were the following: offspring’s CTQ-SF dimensions and total score; the RAAS anxiety and close–depend dimensions; the BSI dimensions and indexes. The exposure variables were the veterans’ war exposure and lifetime PTSD. We estimated two models: the crude model and a model adjusted for the veterans’ respective symptom dimension. Figure 2 illustrates the relationships studied between the exposure variables and the outcome variables. The statistical significance level was fixed at 0.05. Data were analysed using SPSS^®^ software, version 23.

## Results

Table 1 shows the characteristics of the offspring studied sample and their respective fathers. Offspring of fathers with PTSD were not different from offspring of fathers without PTSD, with respect to age, gender, SES, and marital status.

**Table 1:** Sociodemographic characteristics of war veterans and their respective offspring

	War veterans				Offspring			
	Total (*n* = 46)	PTSD (*n* = 27)	Non-PTSD (*n* = 19)	*p*	Total (*n* = 73)	PTSD^c^ (*n* = 43)	Non-PTSD^c^ (*n* = 30)	*p*
Age, years (mean, SD)	65.2, 3.3	64.9, 3.6	65.7, 2.9	0.449	35.7, 4.8	35.6, 4.9	35.8, 4.8	0.835
CAPS score, lifetime (mean, SD)	56.0, 42.8	88.0, 18.2	10.5, 19.3	*<* *0.001*	9.7, 22.9	7.6, 19.9	12.7, 26.7	0.353
Gender	*n* (%)	*n* (%)	*n* (%)		*n* (%)	*n* (%)	*n* (%)	
Female	–	–	–		44 (60.3)	24 (55.8)	20 (66.7)	0.467
Male	–	–	–		29 (39.7)	19 (44.2)	10 (33.3)	
Marital status								
Married/co-habiting^a^	43 (93.5)	26 (96.3)	17 (89.5)	0.561	48 (65.8)	26 (60.5)	22 (73.3)	0.463
Divorced or widowed^b^	3 (6.5)	1 (3.7)	2 (10.5)		5 (6.8)	4 (9.3)	1 (3.3)	
Single	–	–	–		20 (27.4)	13 (30.2)	7 (23.3)	
Graffar								
1	–	–	–		7 (9.6)	3 (7.0)	4 (13.3)	0.206
2	5 (10.9)	2 (7.4)	3 (15.8)	0.613	28 (38.4)	15 (34.9)	13 (46.4)	
3	29 (63.0)	17 (63.0)	12 (63.2)		33 (45.2)	20 (46.5)	13 (43.3)	
4	12 (26.1)	8 (29.6)	4 (21.1)		5 (6.8)	5 (11.6)	0 (0)	
Disability								
No	30 (65.2)	21 (77.8)	9 (47.4)	0.058	–	–	–	
Yes	16 (34.8)	6 (22.2)	10 (52.6)		–	–	–	
Territory (former colony)								
Angola	14 (30.4)	8 (29.6)	6 (31.6)	0.699	–	–	–	
Mozambique	14 (30.4)	7 (25.9)	7 (36.8)		–	–	–	
Guinea	18 (39.1)	12 (44.4)	6 (31.6)		–	–	–	

Italic shows statistically significant results

*SD* standard deviation, *PTSD* posttraumatic stress disorder

^a^For the group of veterans only the married category applies

^b^Widows in the group of veterans, divorced in the group of veterans’ offspring

^c^The offspring were grouped according to their respective fathers’ lifetime PTSD diagnosis

As shown in Table 2, veterans’ war exposure was associated with BSI scores in the offspring with regard to somatisation, phobic anxiety, and GSI and PSDI. None of the other offspring’s variables was associated with veterans’ war exposure. Fathers with lifetime PTSD had no association with any of the offspring’s variables studied.

**Table 2:** Association between fathers’ war exposure and lifetime PTSD with offspring’s psychometric variables

Exposures	War exposure		PTSD, lifetime	
Outcomes	*β* (95% CI)	*p*	*β* (95% CI)	*p*
CTQ-SF				
Emotional abuse	0.076 (− 0.046, 0.197)	0.217	0.271 (− 1.558, 2.100)	0.763
Sexual abuse	0.008 (− 0.083, 0.099)	0.861	− 0.383 (− 1.733, 0.966)	0.564
Physical abuse	0.041 (− 0.019, 0.100)	0.176	0.397 (− 0.485, 1.279)	0.363
Emotional neglect	0.038 (− 0.089, 0.166)	0.548	0.622 (− 1.296, 2.539)	0.511
Physical neglect	0.046 (− 0.003, 0.095)	0.063	0.073 (− 0.717, 0.862)	0.851
Total CTQ-SF	0.199 (− 0.135, 0.532)	0.236	0.792 (− 4.101, 5.686)	0.742
RAAS				
Anxiety	0.009 (− 0.020, 0.039)	0.529	− 0.041 (− 0.506, 0.423)	0.856
Close–depend	− 0.011 (− 0.027, 0.004)	0.145	− 0.167 (− 0.410, 0.076)	0.170
BSI				
Somatisation^a^	*0.025 (0.001*, *0.050)*	*0.043*	0.059 (− 0.298, 0.416)	0.749
Obsessive–compulsive	0.009 (− 0.014; 0.032)	0.442	0.063 (− 0.293, 0.420)	0.718
Interpersonal sen.	0.192 (0.008, 0.053)	0.391	0.131 (− 0.254, 0.515)	0.491
Depression^a^	0.017 (− 0.017, 0.052)	0.319	0.010 (− 0.540, 0.560)	0.971
Anxiety	0.020 (− 0.001, 0.040)	0.056	0.217 (− 0.092, 0.526)	0.160
Hostility^a^	0.010 (− 0.017, 0.038)	0.454	0.231 (− 0.192, 0.654)	0.272
Phobic anxiety	*0.014 (0.000*, *0.027)*	*0.046*	0.043 (− 0.173, 0.259)	0.688
Paranoid ideation^a^	0.008 (− 0.004, 0.021)	0.183	0.109 (− 0.090, 0.308)	0.269
Psychoticism	0.092 (0.002, 0.044)	0.653	0.211 (− 0.118, 0.540)	0.199
GSI	*0.022 (0.005*, *0.038)*	*0.012*	0.138 (− 0.135, 0.411)	0.306
PST	0.278 (− 0.091, 0.647)	0.136	2.823 (− 2.988, 8.633)	0.327
PSDI	*0.020 (0.006*, *0.033)*	*0.006*	0.109 (− 0.119, 0.34)	0.337

Italic shows statistically significant results

*PTSD* posttraumatic stress disorder, *CTQ-SF* Childhood Trauma Questionnaire-Short Form, *RAAS* Revised Adult Attachment Scale, *BSI* Brief Symptom Inventory, *Interpersonal sen.* Interpersonal sensitivity, *GS* Global Severity Index, *PST* Positive Symptom Total, *PSDI* Positive Symptom Distress Index

^a^The variable was log-transformed

After adjusting each significant offspring’s BSI outcome for their respective fathers’ BSI symptom dimension, 18% of the effect of the veterans total war exposure on offspring’s BSI GSI was mediated by the fathers’ BSI GSI (Table 3). For the other BSI scores, the effect of the veterans’ total war exposure on the offspring’s BSI score is direct and is independent of the fathers’ BSI.

**Table 3:** Linear regression coefficients of the association between veterans total war exposure and significant BSI outcomes of their offspring, crude (Model 1), and after adjusting for veterans’ respective symptom dimension (Model 2)

Outcomes	Model 1		Model 2		*β*_2_/*β*_1_ (%)
BSI	*β*_1_	95% CI	*β*_2_	95% CI	
Somatisation	*0.025*	*0.001*, *0.050*	0.024^a^	− 0.004, 0.051	4
Phobic anxiety	*0.014*	*0.000*, *0.027*	0.014^b^	− 0.002, 0.029	0
GSI	*0.022*	*0.005*, *0.038*	*0.018* ^c^	*0.003*, *0.040*	18
PSDI	*0.020*	*0.006*, *0.033*	*0.021* ^d^	*0.004*, *0.039*	− 5

Italic shows statistically significant results

*BSI* Brief Symptom Inventory, *CI* confidence interval, *GSI* Global Severity Index, *PSDI* Positive Symptom Distress Index

^a^Adjusted for the veterans’ somatisation dimension

^b^Adjusted for the veterans’ phobic anxiety dimension

^c^Adjusted for the veterans’ GSI

^d^Adjusted for the veterans’ PSDI

## Discussion

Our study showed that in our sample, veterans’ war exposure is positively associated with BSI scores in the adult offspring regarding somatisation, phobic anxiety, and the general indices of symptomatology GSI and PSDI. For the most part, these associations do not depend on the father’s current psychopathology. Although veterans’ war exposure was not associated with offspring’s self-reported childhood physical neglect, this association was near statistical significance. We found no association between veterans’ lifetime PTSD and offspring psychopathology, attachment dimensions, and self-reported overall childhood maltreatment.

Another study of veterans’ mainly adolescent children’s psychopathology also found that the consequences of combat exposure seem to be more important than those of PTSD [7]. In a study of children aged 6 to 16 years old, Rosenheck and Fontana [34] found that the association between veterans’ participation in abusive violence and child behaviour problems is only minimally mediated by PTSD symptoms. Yet another study, which was carried out 13 years after a war, found an association between veterans’ PTSD and anxiety and aggression among adolescent children [3]. Another study which took place 6 years after a war found higher depression scores among adolescent offspring whose fathers had PTSD [7]. On the other hand, two other studies of mainly young adults did not find any association between veterans’ PTSD and offspring psychopathology [4, 9]. In the sample we studied, it appears that adult offspring may have managed to overcome the burden of having lived with a father with PTSD. As our offspring sample has a high mean age and a higher level of education than their fathers, these factors could have enabled them to adapt and overcome the adverse influences of the consequences of their fathers’ PTSD, but seemingly not the burden of having lived with their father’s experiences of war. One possible explanation for this result is that we studied lifetime PTSD, which means that the PTSD was not necessarily always present. Our study suggests that severity of exposure to war has long-lasting consequences on the next generation’s general psychopathology. The results may suggest that the impact of the fathers’ current psychopathology on the current psychopathology of the adult offspring is limited, with only BSI GSI showing a small possible mediation effect. This could mean that war exposure intensity might have an effect on the offspring’s psychopathology. According to Beckham et al. [14], combat exposure may contribute to interpersonal violence beyond PTSD severity and this could be one of the mechanisms for the intergenerational transmission of war-related trauma.

Glenn et al. [8] found no correlation between war veterans’ combat exposure or PTSD scores and their children’s general psychological distress, including in young adult children. Their sample size was small, and the lack of significance might have been due to lack of power.

Recent research addressing intergenerational transmission of PTSD in a non-war traumatised population did not find an association between parental PTSD and adult offspring’s mental health [23]. One longitudinal study of intergenerational transmission of trauma found lower rates of PTSD and other co-morbid symptoms in later follow-ups of veteran offspring of Holocaust survivors than veteran offspring of non-Holocaust survivors, although the fathers’ traumatic load was not assessed [25]. Another study of older adult offspring of Holocaust survivors found a higher sense of well-being, but more physical problems than offspring of non-Holocaust survivors [27]. Once again, the traumatic experience of the parents was not known. We did not assess physical problems, but found an association between severity of war exposure and BSI symptom cluster somatisation. Recent research which assessed former prisoners of war PTSD with a self-report questionnaire found more secondary traumatic symptoms in adult offspring of former prisoners of war, but no clinical range was reported, neither was significance found [28, 53–56].

Our finding of an association which approximates statistical significance between veterans’ war exposure and their offspring’s self-reported physical neglect is in accordance with other studies of non-war trauma populations, which found high levels of offspring childhood trauma, including physical neglect associated with parent traumatisation during the Holocaust [30]. This could represent a possible mechanism for the intergenerational transmission of war exposure intensity. Yehuda et al. [57] found no increased childhood traumatic experiences among the offspring of Holocaust survivors with PTSD when compared to the offspring of Holocaust survivors without PTSD, or the offspring of non-Holocaust survivors. On the other hand, Dias et al. [5] found that the offspring of war veterans with PTSD self-reported more childhood maltreatment than the offspring of war veterans without PTSD, with no differences between the offspring of veterans without PTSD and the offspring of non-war exposed fathers. Furthermore, they found that offspring’s emotional and physical neglect were predicted by the war status of their fathers. However, fathers’ PTSD diagnosis was differentially assessed across groups, and war exposure was not quantified.

We found no associations between veterans’ lifetime PTSD and war exposure with offspring’s adult attachment. To our knowledge, this is the first study that addresses this issue for the adult offspring of male war veterans. Sagi-Schwartz et al. [58] found that the adult offspring of female Holocaust survivors have secure attachment representations, as do the offspring of non-Holocaust survivors. Evidence exists of an association of mothers’ trauma and PTSD with their 13-month-old child attachment patterns [59]. One recent study found that adult offspring attachment anxiety was higher if their former prisoners of war fathers had PTSD, when compared to those whose fathers did not have PTSD [54]. In this same study, fathers’ PTSD was assessed with a self-report questionnaire and associations of offspring’s attachment orientations and their fathers’ traumatic load were not studied. Adult attachment internal working models are multidetermined and are thought to depend at least in part on the early attachment figures. In our present study, we did not include the mother, which could have had an important buffer role for the offspring under study.

### Strengths and limitations

One important aspect of the present study is that both fathers and children were directly and objectively evaluated. The CAPS gold-standard instrument was used to diagnose veterans’ PTSD. Although the version used in this study was not validated for the Portuguese population, we found that principal components analysis separated contents according to that expected, and that Cronbach’s alpha was according to recommendations (> 0.90). In addition, offspring self-reported their symptoms and the influence of the veterans’ distress in the evaluation of their children symptomatology did not occur as might have happened in other studies [13, 34, 60–62].

It would be advantageous to have included a third group of parents with no experience of war-related TEs and their respective children. This would have given additional information regarding the differential impact of the exposure to war on the next generation. In future studies, mothers should also be included, as they have an important moderating role in the relationship between father and offspring, and their mental health status has a major influence on offspring’s psychopathology [7, 8, 26]. Our results cannot be generalised for the cases of female veterans and non-Caucasians. Sample size might have conditioned the lack of significance of some associations, and the cross-sectional nature of the study precludes any causal inferences. Furthermore, there is a need to study the longitudinal changes in fathers’ and offspring’s psychopathology in order to clarify the dynamics of mediational effects across time. Our results need to be interpreted with caution, due to the possibility of recall bias.

### Management recommendations

Considering the major finding of our research, the significant associations between veterans’ war exposure intensity and their respective offspring’s psychopathological symptoms 40 years after the war ended should be considered when designing veterans’ mental health care systems, which should include providing extended care to the offspring into adulthood. Furthermore, deployed individuals returning from war should be the target of psychoeducational programmes and should be provided with family support care, which takes in account war exposure intensity risk for their offspring’s mental health in the long term.

These findings warrant further research into the intergenerational transmission of trauma.

## Conclusion

Although the sample size may have influenced the consistency of the results, our findings suggest that adult offspring of war veterans are resilient to the consequences of their fathers’ PTSD, but not to those of their fathers’ intensity of war exposure. This latter finding could have considerable social importance and warrants assuming the position that mental health support could benefit the offspring of war veterans as a function of their fathers’ war exposure intensity, and not just of their fathers’ PTSD status. Such intervention could prevent offspring’s psychological suffering, as it could be administered early on after highly traumatised veterans return from war, and not just later on—if and when they develop PTSD.

## Data Availability

The datasets used and analysed for this study are available from the corresponding author on reasonable request.

## Abbreviations

ADFA: Portuguese Disabled Veterans Association
BSI: Brief Symptom Inventory
CAPS: Clinician-Administered PTSD Scale
CI: confidence interval
CTQ-SF: Childhood Trauma Questionnaire-Short Form
DSM-IV: Diagnostic and Statistical Manual of Mental Disorders, Fourth Edition
GSI: Global Severity Index
PSDI: Positive Symptom Distress Index
PST: Positive Symptom Total
PTSD: posttraumatic stress disorder
RAAS: Revised Adult Attachment Scale
SCID-I: Structured Clinical Interview for DSM-IV axis I
SD: standard deviation
SES: socioeconomic status
SPSS: Statistical Package for the Social Sciences
TE: traumatic event
WEQ: War Exposure Questionnaire
